# Type 2 diabetes mellitus and sleep characteristics: a population-based study in Tumbes, Peru

**DOI:** 10.17843/rpmesp.2022.391.10755

**Published:** 2022-03-31

**Authors:** Lucía Ruiz-Burneo, Jimy A. Merino-Rivera, Antonio Bernabé-Ortiz

**Affiliations:** 1 Universidad Científica del Sur, Lima, Peru. Universidad Científica del Sur Universidad Científica del Sur Lima Peru; 2 CRONICAS Centro de Excelencia en Enfermedades Crónicas, Universidad Peruana Cayetano Heredia, Lima, Peru. Universidad Peruana Cayetano Heredia CRONICAS Centro de Excelencia en Enfermedades Crónicas Universidad Peruana Cayetano Heredia Lima Peru

**Keywords:** Sleep, Sleep Hygiene, Sleep Quality, Type 2 Diabetes Mellitus

## Abstract

**Objective.:**

To determine if there is an association between type 2 diabetes *mellitus* (T2DM) and some sleep characteristics: duration, sleep difficulties and quality, in a population aged between 30 and 69 years in Tumbes.

**Materials and methods.:**

Cross-sectional study. The outcomes were sleep difficulty (sometimes/ almost never vs. frequently), sleep duration (normal, short, and long), and sleep quality (good and bad). The exposure of interest was the presence of T2DM assessed using the glucose tolerance test (without T2DM, with T2DM but without previous diagnosis, and with T2DM and with previous diagnosis). Poisson regression models were used to report prevalence ratio (PR) and 95% confidence intervals (95%CI).

**Results.:**

A total of 1604 subjects were analyzed and the mean age was 48.2 years; 50.3% were women, 71 (4.4%) had T2DM without a previous diagnosis, and 105 (6.5%) had T2DM with a previous diagnosis. Regarding sleep characteristics, 12.0% had short sleep, 8.2% had long sleep, 3.7% had sleep difficulties, and 19.5% presented poor sleep quality. In a multivariable model, T2DM with previous diagnosis was associated with sleep difficulty (PR= 2.20; 95%CI: 1.13 - 4.27) and bad sleep quality (PR= 1.40; 95%CI: 1.05 - 1.92) compared to those without T2DM.

**Conclusions.:**

Individuals with previous diagnosis of T2DM had greater probability of presenting sleep difficulties and poor sleep quality. These results suggest the need to evaluate periodically the sleep characteristics of patients with T2DM.

## INTRODUCTION

Among current health challenges, noncommunicable diseases are those that affect most of the world’s population, with the emphasis on cardiovascular and respiratory conditions as well as type 2 diabetes *mellitus* (T2DM). The latter, according to World Health Organization (WHO) projections, is expected to become the seventh leading cause of death by 2030 ^1^. T2DM is a chronic disease characterized by high blood glucose levels. In 1980, the number of adults suffering from this disease was 108 million globally, and according to projections, by 2030 the figure will increase to 578 million ^2^, and by 2045, the figure will be 700 million ^3^.

T2DM can affect different organs and body functions, and several studies have shown a significant association with changes in sleep patterns. For example, a relatively recent systematic review reported that the prevalence of sleep disorders in T2DM was high (52%) ^4^. Similar results were found in Spain where a study using the Pittsburgh Sleep Quality Index (PSQI) reported that more than half of patients with T2DM had poor sleep quality ^5^. On the other hand, a study in Japan evaluated the quality and duration of sleep among patients with T2DM, finding a higher prevalence of short sleep and poor sleep quality in those with higher levels of glycosylated hemoglobin ^6^.

Although sleep has several characteristics, few studies have evaluated the association between T2DM and these characteristics in Latin America. For example, in Venezuela, 95.5% of patients with T2DM were found to have poor sleep quality ^7^, although the results were merely descriptive. Most previous studies focus on the presence of sleep apnea in subjects with obesity and its impact on the development of T2DM ^8^. In the case of Peru, the prevalence of T2DM has been estimated at 7% ^9^, with marked variations in the different regions, being higher in the north of the country. Thus, a study in Tumbes determined that the prevalence of T2DM was as high as 10% ^10^. On the other hand, it is estimated that Peruvians sleep an average of 7.7 hours on weekdays and up to 8 hours on weekends, while the prevalence of short and long sleep is 4.3% and 22.4%, respectively ^11^. However, whether the high prevalence of T2DM may affect existing sleep patterns in the population is unknown.

Therefore, the aim of this study was to determine whether there is an association between T2DM and certain sleep characteristics, particularly sleep duration, sleep difficulty, and sleep quality in Tumbes, Peru.

**KEY MESSAGES**

Motivation for the study: According to current evidence, type 2 diabetes *mellitus* (DM2) may have an effect on certain sleep characteristics. However, information is limited in countries such as Peru.

Main findings: Of the evaluated total, 12.0% had short sleep, 8.2% had long sleep, 3.7% had difficulty sleeping, and 19.5% had poor sleep quality. Having DM2 with previous diagnosis was associated with a higher probability of having difficulty sleeping and poor sleep quality.

Implications: Our results suggest the need to periodically evaluate sleep characteristics in patients with DM2.

## MATERIALS AND METHODS

### Study design

Secondary analysis of data from a population-based cross-sectional study conducted in Tumbes to evaluate different methods of screening for T2DM ^12^. The original study aimed to evaluate the diagnostic accuracy of the Finnish Diabetes Risk Score (FINDRISC) for T2DM case detection and to compare its diagnostic performance against the oral glucose tolerance test.

### Study population

The population included in the original study consisted of permanent residents of Tumbes (≥ 6 months) between 30 and 69 years of age, who were capable of understanding the procedures and giving informed consent. Pregnant women, persons unable to answer questionnaires, bedridden, with active TB, Alzheimer’s, Parkinson’s or persons with any physical disability that prevented anthropometric measurements (weight, height, blood pressure or waist circumference) were excluded ^12^.

The inclusion criteria of the original study were also used for our analysis. However, in this case we considered only those with complete information on the variables of interest, i.e., the oral glucose tolerance test to define T2DM and the sleep characteristics assessed.

### Sampling

The original study used randomized, sex-stratified sampling using the most recent study area census (conducted in 2014). Only one person per household was selected to avoid clustering of risk factors.

### Sample size

To ensure that this study adequately assesses the association between variables, we applied power calculation using Open EPI (Open Source Epidemiological Statistics for Public Health) software. Assuming a 95% confidence level and a prevalence of poor sleep quality in unexposed (those without T2DM) of 18.5% and a prevalence ratio of 1.67, we concluded that with data from 1604 participants we would achieve a power of 87% to find association between the variables of interest.

### Variables

*Dependent variable*

The dependent variables of interest were three, and were related to certain sleep characteristics:

Sleep duration: evaluated with the following question: “*On average, in the last year, how many hours did you sleep in a day?*”, and categorized according to the definition of the National Sleep Foundation (NSF) of the United States ^13^: normal duration of sleep between 7 and 9 h if the person was between 26 and 64 years old, or between 7 and 8 h if the person was 65 years old or older ^14^. Less hours than those were considered as short sleep, and more hours were considered as long sleep.

Sleep quality: Sleep quality was assessed using the Pittsburgh Sleep Quality Index (PSQI), which is an instrument consisting of a 21-question questionnaire. Questions are divided into seven modules: subjective sleep quality, duration, sleep disturbance, latency, use of sleep medication, sleep efficiency and daytime dysfunction ^15^. These seven components are evaluated resulting in an overall score that can vary between 0 and 21 points. Poor sleep quality is defined with a score >5. In 2015, this instrument was validated for Peruvian population using a sample of 4445 people ^16^.

Difficulty sleeping was assessed through the question: “During the last month, have you had difficulty sleeping?”, with three response options: “almost never”, “sometimes”, and “frequently”. For analysis, this variable was collapsed by putting together the first two response options (almost never/sometimes vs. frequently).

*Independent variable*

The independent variable was glycemic status, with emphasis on T2DM. As defined by the American Diabetes Association (ADA) standards using the oral glucose tolerance test ^17^: participants with T2DM were those with fasting plasma glucose values ≥126 mg/dL or postprandial (at 2 h) glucose values ≥200 mg/dL or those who reported having previous diagnosis of such condition (yes vs. no). For analysis purposes and based on previous reports ^18^, persons with the presence of T2DM were subdivided into two categories based on whether or not they had previous diagnosis. Thus, the final variable had three categories: without T2DM; with T2DM, but without previous diagnosis; and with T2DM and previous diagnosis.

*Other variables*

The other variables used in the analysis were: sex (male vs. female); age (<50 vs. ≥50 years); level of education (<7 years, 7-11 years, and ≥12 years); socioeconomic status, assessed by home possessions and then classified into three categories (low, middle, and high); whether currently working (yes vs. no); daily smoking (yes vs. no); alcohol consumption (≤1 time per month vs. >1 time per month); level of physical activity, assessed through the short version of the International Physical Activity Questionnaire (IPAQ) and categorized into moderate/high vs. low; body mass index (normal, overweight, and obese), defined according to traditional cut points; and arterial hypertension (yes vs. no), defined according to the presence of systolic blood pressure ≥140 mm Hg, or diastolic blood pressure ≥90 mm Hg, or the report of previous diagnosis made by a physician.

### Procedures

The procedures of the original study have been detailed previously ^12^. Briefly, after informed consent was obtained, participants were summoned on fasting for the oral glucose tolerance test. Blood samples were obtained by trained health care personnel. Venous blood was drawn from the participants, who were required to have been fasting for 8 to 12 hours before the first sample was taken, whereas the second sample was taken at 2 hours post-ingestion of 75 g of anhydrous glucose dissolved in 300 mL of water ^17^. The questionnaires and clinical and anthropometric measurements were performed during the time between the two blood sampling times.

The questionnaires were administered by means of tablet surveys, using Open Data Kit software, and were conducted by trained health personnel. Questionnaires included information on risk scores for T2DM, in addition to other sociodemographic and lifestyle details.

Subsequently, height (using a calibrated measuring rod), weight with a bioimpedance scale (TANITA TBF-300A) and waist circumference (in cm) were measured in triplicate. Blood pressure was also measured using an OMRON HEM-780 automatic monitor in triplicate, after 5 min of rest (the average of the last two measurements was used to determine the presence of hypertension).

### Statistical analysis

STATA version 16 for Windows (StataCorp, College Station, TX, USA) was used for the analysis. First, the study population was characterized using means and standard deviation (SD) for numerical variables, and proportions for categorical variables. We also estimated the prevalence of sleep duration (normal, short, and long), poor sleep quality, and difficulty sleeping, as well as the prevalence of T2DM in the study population. Comparisons of the different sociodemographic and behavioral variables according to the presence of T2DM and sleep characteristics were carried out using the chi-square test.

Finally, we created crude and adjusted models using Poisson regression models with robust variance to assess whether the associations of interest existed. Potential confounders were chosen according to an epidemiological model based on the existing literature. Prevalence ratios (PR) and 95% confidence intervals (95% CI) were reported.

### Ethical Aspects

The original study protocol and the consent forms and questionnaires were approved by the Ethics Committee of the Universidad Peruana Cayetano Heredia, Lima, and by the Institutional Ethics Committee of the London School of Hygiene and Tropical Medicine, London. For this study, the data were coded to guarantee the anonymity of the participants. Approval was obtained from the ethics committee of the Universidad Científica del Sur (137-2020-PRE15).

## RESULTS

### Characteristics of the study population

A total of 1612 individuals were enrolled, but 3 (0.2%) participants did not complete the oral glucose tolerance test examinations and 5 (0.3%) did not have complete data on sleep-related variables. Therefore, only 1604 individuals were analyzed. The mean age of the entire sample was 48.2 years (SD: 10.6); 809 (50.3%) were women; 73.5% were overweight or obese, and 67.8% were employed at the time of the study.

### Type 2 diabetes *mellitus* and associated factors

Of the 1604 individuals analyzed, 175 (10.9%; 95% CI: 9.4% - 12.5%) had T2DM, and of the latter, 71 (4.4%) did not know they had this condition, while 105 (6.5%) had previous diagnosis. Of the evaluated variables during bivariate analysis, age (p<0.001), level of education (p<0.001), being currently working (p=0.001), level of physical activity (p=0.005), body mass index (p=0.023), and the presence of hypertension (p<0.001) were associated with T2DM (Table 1).

**Table 1:** Characteristics of the study population, according to glycemic status.

Characteristic	Without T2DM	T2DM without previous diagnosis	T2DM with previous diagnosed	p-value
	(n = 1429)	(n = 71)	(n = 104)	
Sex				
Male	724 (50.7%)	29 (40.9%)	44 (42.3%)	0.081
Female	705 (49.3%)	42 (59.1%)	60 (57.7%)	
Age				
< 50 years	859 (60.1%)	36 (50.7%)	23 (22.1%)	<0.001
≥ 50 years	570 (39.9%)	35 (49.3%)	81 (77.9%)	
Education level				
< 7 years	438 (30.7%)	27 (38.0%)	52 (50.0%)	<0.001
7 - 11 years	673 (47.1%)	32 (45.1%)	41 (39.4%)	
≥ 12 years	318 (22.2%)	12 (16.9%)	11 (10.6%)	
Socioeconomic status				
Low	469 (32.8%)	25 (35.2%)	42 (40.4%)	0.430
Middle	496 (34.7%)	20 (28.2%)	33 (31.7%)	
High	464 (32.5%)	26 (36.6%)	29 (27.9%)	
Currently working				
No	439 (30.7%)	29 (40.9%)	49 (47.1%)	0.001
Yes	990 (69.3%)	42 (59.1%)	55 (52.9%)	
Daily smoking				
No	1345 (94.1%)	69 (97.2%)	98 (94.2%)	0.556
Yes	84 (5.9%)	2 (2.8%)	6 (5.8%)	
Alcohol use				
≤ 1 time per month	1287 (90.1%)	63 (88.7%)	101 (97.1%)	0.054
>1 time per month	142 (9.9%)	8 (11.3%)	3 (2.9%)	
Physical activity level				
Moderate/high	909 (63.6%)	44 (62.0%)	49 (47.1%)	0.004
Low	520 (36.4%)	27 (38.0%)	55 (52.9%)	
Body mass index				
Normal	388 (27.2%)	11 (15.5%)	26 (25.0%)	0.023
Overweight	625 (43.7%)	28 (39.4%)	52 (50.0%)	
Obese	416 (29.1%)	32 (45.1%)	26 (25.0%)	
Hypertension				
No	1084 (75.9%)	46 (64.8%)	58 (57.8%)	<0.001
Yes	345 (24.1%)	25 (35.2%)	46 (44.2%)	

T2DM: type 2 diabetes *mellitus*

### Sleep characteristics and associated factors

Regarding sleep duration, the study population reported sleeping an average of 7.8 h (SD: 1.2); 12.0% (95% CI: 10.5% - 13.7%) of subjects had short sleep, while 8.2% (95% CI: 6.9% - 9.6%) had long sleep. Age (p=0.012), sex (p<0.001), education level (p=0.030), socioeconomic status (p=0.007), being currently working (p<0.001), daily smoking (p=0.001), physical activity level (p= 0.003), and body mass index (p=0.042) were variables associated with sleep duration in bivariate analysis (Table 2).

**Table 2:** Characteristics of the study population, according to sleep duration.

Characteristics	Sleep duration			p-value
	Normal sleep	Short sleep	Long sleep	
	(n = 1280)	(n = 193)	(n = 131)	
Sex				
Male	627 (49.0%)	120 (62.2%)	50 (38.2%)	<0.001
Female	653 (51.0%)	73 (37.8%)	81 (61.8%)	
Age				
< 50 years	756 (59.1%)	95 (49.2%)	67 (51.2%)	0.012
≥ 50 years	524 (40.9%)	98 (50.8%)	65 (48.8%)	
Education level				
< 7 years	399 (31.2%)	68 (35.2%)	50 (38.2%)	0.030
7 - 11 years	606 (47.3%)	76 (39.4%)	64 (48.9%)	
≥ 12 years	275 (21.5%)	49 (25.4%)	17 (12.9%)	
Socioeconomic status				
Low	430 (33.6%)	50 (25.9%)	56 (42.8%)	0.007
Middle	450 (35.2%)	66 (34.2%)	33 (25.2%)	
High	400 (31.2%)	77 (39.9%)	42 (32.0%)	
Currently working				
No	411 (32.1%)	42 (21.8%)	64 (48.9%)	<0.001
Yes	869 (67.9%)	151 (78.2%)	67 (51.1%)	
Daily smoking				
No	1215 (94.9%)	171 (88.6%)	126 (96.2%)	0.001
Yes	65 (5.1%)	22 (11.4%)	5 (3.8%)	
Alcohol use				
≤ 1 time per month	1160 (90.6%)	170 (88.1%)	121 (92.4%)	0.395
>1 time per month	120 (9.4%)	23 (11.9%)	10 (7.6%)	
Physical activity level				
Moderate/high	803 (62.7%)	133 (68.9%)	66 (50.4%)	0.003
Low	477 (37.3%)	60 (31.1%)	65 (49.6%)	
Body mass index				
Normal	347 (27.1%)	42 (21.8%)	36 (27.5%)	0.042
Overweight	573 (44.8%)	87 (45.1%)	45 (34.4%)	
Obese	360 (28.1%)	64 (33.1%)	50 (38.1%)	
Hypertension				
No	953 (74.5%)	142 (73.6%)	93 (71.0%)	0.681
Yes	327 (25.5%)	51 (26.4%)	38 (29.0%)	
Glycemic status				
No T2DM	1141 (89.1%)	173 (89.6%)	115 (87.8%)	0.377
T2DM without previous diagnosis	60 (4.7%)	8 (4.2%)	3 (2.3%)	
T2DM with previous diagnosis	79 (6.2%)	12 (6.2%)	13 (9.9%)

T2DM: type 2 diabetes *mellitus*

On the other hand, 3.7% (95% CI: 2.8% - 4.7%) reported difficulty sleeping, while 19.5% (95% CI: 17.5% - 21.5%) of subjects had poor sleep quality. During bivariate analysis, sex (p=0.027), age (p= 0.019), education level (p=0.035), and being currently working (p=0.002) were associated with difficulty sleeping; while age (p<0.001), sex (p=0.002), education level (p<0.001), being currently working (p<0.001), and presence of hypertension (p=0.006) were associated with poor sleep quality (Table 3).

**Table 3:** Characteristics of the study population, according to sleep difficulty and sleep quality.

Characteristics	Sleep difficulty		p-value	Sleep quality		p-value
	No	Yes		Good	Poor	
	(n = 1545)	(n = 59)		(n = 1292)	(n = 312)	
Sex						
Male	776 (50.2%)	21 (35.6%)	0.027	683 (52.9%)	114 (36.5%)	<0.001
Female	769 (49.8%)	38 (64.4%)		609 (47.1%)	198 (63.5%)	
Age						
< 50 years	893 (57.8%)	25 (42.4%)	0.019	768 (59.4%)	150 (48.1%)	<0.001
≥ 50 years	652 (42.2%)	34 (57.6%)		524 (40.6%)	162 (51.9%)	
Education level						
< 7 years	489 (31.7%)	28 (47.5%)	0.035	387 (30.0%)	130 (41.7%)	<0.001
7 - 11 years	726 (47.0%)	20 (33.9%)		622 (48.1%)	124 (39.7%)	
≥ 12 years	330 (21.3%)	11 (18.6%)		283 (21.9%)	58 (18.6%)	
Socioeconomic status						
Low	514 (33.3%)	22 (37.3%)	0.502	425 (32.9%)	111 (35.6%)	0.665
Middle	533 (34.5%)	16 (27.1%)		446 (34.5%)	103 (33.0%)	
High	498 (32.2%)	21 (35.6%)		421 (32.6%)	98 (31.4%)	
Currently working						
No	487 (31.5%)	30 (50.9%)	0.002	391 (30.3%)	126 (40.4%)	<0.001
Yes	1058 (68.5%)	29 (49.1%)		901 (69.7%)	186 (59.6%)	
Daily smoking						
No	1456 (94.2%)	56 (94.9%)	0.827	1221 (94.5%)	291 (93.3%)	0.400
Yes	89 (5.8%)	3 (5.1%)		71 (5.5%)	21 (6.7%)	
Alcohol use						
≤ 1 time per month	1395 (90.3%)	56 (94.9%)	0.235	1168 (90.4%)	283 (90.7%)	0.870
>1 time per month	150 (9.7%)	3 (5.1%)		124 (9.6%)	29 (9.3%)	
Physical activity level						
Moderate/high	966 (62.5%)	36 (61.0%)	0.814	804 (62.2%)	198 (63.5%)	0.687
Low	579 (37.5%)	23 (39.0%)		488 (37.8%)	114 (36.5%)	
Body mass index						
Normal	417 (27.0%)	8 (13.6%)	0.071	358 (27.7%)	67 (21.5%)	0.076
Overweight	675 (43.7%)	30 (50.8%)		561 (43.4%)	144 (46.1%)	
Obese	453 (29.3%)	21 (35.6%)		373 (28.9%)	101 (32.4%)	
Hypertension						
No	1149 (74.4%)	39 (66.1%)	0.155	976 (75.5%)	212 (68.0%)	0.006
Yes	396 (25.6%)	20 (33.9%)		316 (24.5%)	100 (32.0%)	
Glycemic status						
No T2DM	1362 (89.4%)	47 (79.6%)	0.004	1165 (90.2%)	264 (84.6%)	0.007
T2DM without previous diagnosis	69 (4.5%)	2 (3.4%)		55 (4.2%)	16 (5.1%)	
T2DM with previous diagnosis	94 (6.1%)	10 (17.0%)		72 (5.6%)	32 (10.3%)	

T2DM: type 2 diabetes *mellitus*

### Association between type 2 diabetes *mellitus* and sleep characteristics.

In the multivariate model (Table 4), adjusted for age, sex, level of education, socioeconomic status, being currently working, daily smoking, alcohol use, level of physical activity and presence of hypertension, the presence of previously diagnosed T2DM increased the prevalence of sleep difficulty (PR = 2.20; 95% CI: 1.13 - 4.27) and poor sleep quality (PR = 1.40; 95% CI: 1.05 - 1.92) compared to those without T2DM. On the other hand, no association was found between T2DM and sleep duration.

**Table 4:** Association between glycemic status and sleep: crude and adjusted models.

Sleep characteristics	Crude model	Adjusted model ^a^
	PR (95% CI)	PR (95% CI)
Sleep duration		
Short sleep vs. normal		
No T2DM	1 (Reference)	1 (Reference)
T2DM without previous diagnosis	0.89 (0.46 - 1.74)	0.91 (0.46 - 1.80)
T2DM with previous diagnosis	1.00 (0.58 - 1.73)	0.96 (0.55 - 1.65)
Long sleep vs. normal		
No T2DM	1 (Reference)	1 (Reference)
T2DM without previous diagnosis	0.52 (0.17 - 1.59)	0.44 (0.14 - 1.34)
T2DM with previous diagnosis	1.54 (0.91 - 2.63)	1.14 (0.68 - 1.94)
Sleep difficulty		
No T2DM	1 (Reference)	1 (Reference)
T2DM without previous diagnosis	0.86 (0.21 - 3.46)	0.70 (0.17 - 2.85)
T2DM with previous diagnosis	2.92 (1.52 - 5.62)	2.20 (1.13 - 4.27)
Sleep quality		
No T2DM	1 (Reference)	1 (Reference)
T2DM without previous diagnosis	1.22 (0.78 - 1.90)	1.08 (0.70 - 1.67)
T2DM with previous diagnosis	1.67 (1.22 - 2.27)	1.40 (1.05 - 1.92)

T2DM: type 2 diabetes mellitus; PR: prevalence ratio; 95% CI: 95% confidence interval.

a Adjusted for age, sex, level of education, socioeconomic status, currently working, daily smoking, alcohol use, level of physical activity, and hypertension.

## DISCUSSION

According to this study, individuals previously diagnosed with T2DM had a higher probability of having difficulty sleeping (120% increase), and a higher prevalence of poor sleep quality (40% increase), compared with those without T2DM. The presence of T2DM without previous diagnosis (i.e., those with recent diagnosis) was not associated with any sleep characteristics. On the other hand, one in ten participants was found to have T2DM.

Some studies have found an association between T2DM and certain sleep characteristics. Although we did not find an association between T2DM and sleep duration, a systematic review that only included studies in China found that the prevalence of short sleep, defined as that <6 hours, was 23% in persons with T2DM compared to 12% in healthy persons ^19^. On the other hand, a study using data from nine countries (Finland, Poland, Spain, China, Ghana, India, Mexico, Russia, and South Africa) found an association between T2DM and sleep problems globally, but in the analysis by country, the association was present only in India ^20^. The latter study defined T2DM on the basis of self-report and, therefore, may resemble our group with T2DM and previous diagnosis. However, the variable “sleep problems” was not assessed comprehensively as it was only measured using one question. Another study found that those with T2DM had higher rates of insomnia, excessive sleepiness, and tended to use more hypnotics than the controls ^21^. Finally, a study in Swedish men reported greater difficulties in initiating and maintaining sleep in patients with T2DM than in subjects without this condition ^22^. All these results suggest that T2DM may alter certain sleep characteristics, especially those associated with sleep duration and the presence of difficulties in falling asleep.

It is important to highlight that T2DM, in addition to causing direct sleep disturbances as a consequence of nocturia, polyuria, diabetic neuropathy and pain due to neuropathy, has also been associated with diseases such as obstructive sleep apnea, hypertension, cardiovascular complications, cerebrovascular accidents, obesity, irritability, excessive thirst and even depression, which can alter sleep ^23^. In turn, sleep disturbances in diabetics increase the release of glucocorticoids producing an increase in glucose production and a consequent inefficient glycemic control ^24^ ^,^ ^25^.

Although in Peru there is the “Clinical Practice Guideline for the Diagnosis, Treatment and Control of Diabetes Mellitus type 2 in the First Level of Care” ^26^, it is important to mention that currently this guideline does not contemplate that the population with T2DM could have sleep disturbances. Our study found an association between T2DM with previous diagnosis and poor sleep quality, implying a high prevalence of poor sleep quality in those with T2DM in the population, for whom appropriate management has not been detailed. A meta-analysis of cohort studies reported that sleep disturbances in patients with T2DM lead to a series of complications such as poor glycemic control, glucose intolerance, and insulin resistance ^25^, altered immune system homeostasis and cardiovascular disease ^27^, and even increased mortality ^28^.

Given our results, the current guidelines should include practical tools that take into account simple questions to assess sleep characteristics, since they could be very useful to screen for sleep disturbances and improve the management of patients with T2DM. After an initial evaluation, more complex tools could be considered, if necessary. Interconsultations to specialties could be necessary to ensure a comprehensive management of the patient with T2DM.

Finally, we identified that Tumbes is a city with a high prevalence of T2DM compared to the national average ^9^, and therefore is necessary to identify and understand the factors associated with these differences in order to design public health measures aimed at controlling, preventing and promoting the health of this population. This is a population-based study that used the oral glucose tolerance test to define T2DM, which is considered the gold standard for the diagnosis of this condition. Despite its strengths, this research has some limitations that should be mentioned. First, being a cross-sectional study, only association can be analyzed, but not causality. Second, recall bias may be present, given that several of the factors evaluated are subjective and asked with respect to the previous year or the 30 days prior to the evaluation date, despite the use of validated scales such as the Pittsburgh scale, which are highly reliable ^29^. Likewise, the results cannot be extrapolated to the entire Peruvian population because a region with a high prevalence of T2DM was chosen for this study. Third, there is the possibility of reverse causality given that alterations in sleep characteristics could also cause T2DM as has been described in other studies ^30^ ^,^ ^31^. However, sleep characteristics were assessed with respect to the 30 days prior to the date of the interview, which establishes that it is likely that T2DM occurred first. Fourth, only one glucose tolerance test was used to define T2DM when the usual, according to the ADA, is to use two tests at different times ^17^. Finally, our multivariate models were adjusted for the existing variables in the database, leaving aside other important variables such as time of disease, presence of complications (coronary artery disease, peripheral vascular disease, retinopathy, neuropathies, nephropathy, etc.), hypercholesterolemia, coffee consumption, among others ^32^ ^-^ ^34^. However, our findings are in line with results from previous studies.

In conclusion, individuals with T2DM who had previous diagnosis were more likely to have difficulty sleeping and poor sleep quality. Our results suggest the need for periodic evaluation of sleep characteristics in patients with T2DM.
