# Supplementary material for: Type 2 diabetes mellitus and sleep characteristics: a population-based study in Tumbes, Peru
Source: Rev Peru Med Exp Salud Publica. 2022 Mar 31;39(1):55–64. doi: 10.17843/rpmesp.2022.391.10755 (PMC11397782; doi:10.17843/rpmesp.2022.391.10755)
Supplement: Translation

# Translation: Diabetes mellitus tipo 2 y características del sueño: un estudio poblacional en Tumbes, Perú

**Authors:** Lucía Ruiz-Burneo, Jimy A. Merino-Rivera, Antonio Bernabé-Ortiz

**Affiliations:** 1 Universidad Científica del Sur, Lima, Perú.; 2 CRONICAS Centro de Excelencia en Enfermedades Crónicas, Universidad Peruana Cayetano Heredia, Lima, Perú.

**Keywords:** Sueño, Higiene del Sueño, Calidad de Sueño, Diabetes Mellitus tipo 2

## Abstract

**Objetivo.:**

Determinar si existe asociación entre la presencia de diabetes *mellitus* tipo 2 (DM2) y la duración, dificultad para dormir, y calidad de sueño, en sujetos de 30 a 69 años en Tumbes.

**Materiales y métodos.:**

Estudio transversal analítico. Las variables dependientes fueron dificultad para dormir (a veces/casi nunca y frecuentemente), duración del sueño (normal, corto y prolongado) y calidad del sueño (buena y mala). La variable independiente fue la presencia de DM2 evaluada usando la prueba de tolerancia oral a la glucosa (no DM2, con DM2 y sin diagnóstico previo, y con DM2 y diagnóstico previo). Se usó modelos de regresión de Poisson para reportar razones de prevalencia (RP) e intervalos de confianza al 95% (IC 95%).

**Resultados.:**

Se analizaron 1604 individuos, con una edad media de 48,2 años; 50,3% fueron mujeres; 71 (4,4%) tenían DM2 sin diagnóstico previo y 105 (6,5%) tenían DM2 con diagnóstico previo. Según las características del sueño, 12,0% presentó sueño corto y 8,2% presentó sueño prolongado; 3,7% reportó dificultad para dormir, y 19,5% tuvo mala calidad de sueño. En el modelo multivariable, tener DM2 con diagnóstico previo estuvo asociado con dificultad para dormir (RP = 2,20; IC 95%: 1,13-4,27) y mala calidad de sueño (RP = 1,40; IC 95%: 1,05-1,92) comparado con aquellos sin DM2.

**Conclusiones.:**

Los individuos con DM2 que tenían diagnóstico previo tuvieron mayor probabilidad de presentar dificultad para dormir y mala calidad de sueño. Nuestros resultados sugieren la necesidad de evaluar en forma periódica las características del sueño en pacientes con DM2.

## INTRODUCCIÓN

Dentro de los retos actuales en salud, las enfermedades no transmisibles son las que más afectan a la población mundial, estando el énfasis en las condiciones cardiovasculares, respiratorias y la diabetes *mellitus* tipo 2 (DM2). Esta última, según proyecciones de la Organización Mundial de la Salud (OMS), se convertiría en la séptima causa de muerte para el 2030 ^1^. La DM2 es una enfermedad crónica caracterizada por niveles altos de glucosa en sangre. En 1980, la cantidad de población adulta que padecía esta enfermedad era de 108 millones a nivel global, y según proyecciones, para el año 2030 la cifra aumentará a 578 millones ^2^, y para el 2045, la cifra será de 700 millones ^3^.

La DM2 puede afectar distintos órganos y funciones corporales, y varios estudios han evidenciado una asociación significativa con cambios en los patrones del sueño. Por ejemplo, una revisión sistemática relativamente reciente reportó que la prevalencia de desórdenes del sueño en DM2 era alta (52%) ^4^. Resultados similares se encontraron en España donde un estudio usando el Índice de Calidad de Sueño Pittsburgh (PSQI de sus siglas en inglés) reportó que más de la mitad de pacientes con DM2 presentaron mala calidad de sueño ^5^. De otro lado, un estudio en Japón evaluó la calidad y duración de sueño en pacientes con DM2, encontrando una mayor prevalencia de sueño corto y mala calidad de este en aquellos con mayores niveles de hemoglobina glicosilada ^6^. 

Aunque existen diversas características del sueño, son pocos los estudios que han evaluado la asociación entre DM2 y dichas características en América Latina. Por ejemplo, en Venezuela se encontró que 95,5% de los pacientes con DM2 presentaban mala calidad de sueño ^7^, aunque los resultados fueron meramente descriptivos. La mayoría de trabajos previos se enfocan en la presencia de apnea-sueño en sujetos con obesidad y su impacto en el desarrollo de DM2 ^8^. En el caso de Perú, la prevalencia de DM2 ha sido estimada en 7% ^9^, con variaciones marcadas en las distintas regiones, siendo más alta en el norte del país. Así, un estudio en Tumbes determinó que la prevalencia de DM2 llegaba a 10% ^10^. De otro lado, se estima que los peruanos duermen 7,7 h en promedio los días de semana y hasta 8 h los fines de semana, mientras que la prevalencia de sueño corto y prolongado es de 4,3 y 22,4%, respectivamente ^11^. Sin embargo, si la alta prevalencia de DM2 puede afectar los patrones de sueño existente a nivel poblacional es algo que se desconoce.

Como consecuencia, el objetivo del presente estudio fue determinar si existe asociación entre DM2 y ciertas características del sueño, enfocándose especialmente en la duración, dificultad para dormir, y calidad del sueño en Tumbes, Perú.

**MENSAJES CLAVE**

Motivación para hacer el estudio: de acuerdo con la evidencia actual, la diabetes *mellitus *tipo 2 (DM2) puede tener efecto sobre ciertas características del sueño. Sin embargo, la información es limitada en países como el Perú.

Principales hallazgos: del total evaluado, 12,0% presentaron sueño corto, 8,2% tuvo sueño prolongado, 3,7% presentó dificultad para dormir, y 19,5% tuvo mala calidad de sueño. Tener DM2 con diagnóstico previo estuvo asociado a mayor probabilidad de tener dificultad para dormir y mala calidad de sueño.

Implicancias: nuestros resultados sugieren la necesidad de evaluar periódicamente las características del sueño en pacientes con DM2.

## MATERIALES Y MÉTODOS

### Diseño del estudio

Análisis secundario de datos de un estudio transversal de base poblacional realizado en Tumbes para evaluar diferentes métodos de tamizaje de DM2 ^12^. El estudio original tuvo como objetivo evaluar la precisión diagnóstica del *Score* de Riesgo Finlandés para Diabetes (FINDRISC, de sus siglas en inglés), para la detección de casos de DM2, y comparar su rendimiento diagnóstico contra la prueba de tolerancia oral a la glucosa.

### Población de estudio

La población que se incluyó en el estudio original estuvo conformada por aquellos entre 30 y 69 años, residentes permanentes en Tumbes (≥ 6 meses) y capaces de comprender los procedimientos y dar su consentimiento informado. Se excluyó a las mujeres gestantes, personas no capaces de contestar cuestionarios, postrados en cama, con TBC activa, Alzheimer, Parkinson o personas con alguna discapacidad física que impidiera las mediciones antropométricas (peso, talla, presión arterial o circunferencia de la cintura) ^12^.

Para el presente análisis se utilizaron los mismos criterios de inclusión del estudio original. Sin embargo, en este caso solo se consideró a aquellos que tenían información completa en las variables de interés, es decir, la prueba de tolerancia oral a la glucosa para definir DM2 y las características del sueño evaluadas.

### Muestreo

El estudio original siguió un muestreo aleatorizado y estratificado por sexo usando el censo del área de estudio más actualizado (realizado en el 2014). Se seleccionó a una sola persona por vivienda para evitar la aglomeración de factores de riesgo.

### Tamaño de muestra

Para garantizar que este estudio evalúe adecuadamente la asociación entre las variables, se realizó el cálculo del poder estadístico utilizando el *software* Open EPI (Estadísticas Epidemiológicas de Código Abierto para Salud Pública). Asumiendo un nivel de confianza del 95% y una prevalencia de mala calidad de sueño en no expuestos (aquellos sin DM2) del 18,5% y una razón de prevalencia de 1,67, concluimos que con los datos de 1604 participantes se tiene un poder de 87% para encontrar asociación entre las variables de interés.

### Variables

*Variable dependiente*

Tres fueron las variables dependientes de interés, relacionada con ciertas características del sueño:

Duración de sueño: evaluada a través de la pregunta: *«¿En promedio, en el último año, cuántas horas durmió en un día? »*, y categorizada de acuerdo con la definición de la Fundación Nacional del Sueño (NSF de sus siglas en inglés) de los Estados Unidos ^13^: sueño de duración normal entre 7 y 9 h si la persona tenía entre 26 y 64 años, o entre 7 y 8 h si la persona tenía 65 años o más ^14^. Por debajo de esos valores se consideró sueño corto, mientras que, por encima, se consideró la duración como sueño prolongado. 

Calidad de sueño: Se valoró mediante el índice de calidad de sueño de Pittsburgh (Pittsburgh Sleep Quality Index o PSQI), el cual es un instrumento que consiste en un cuestionario de 21 preguntas. Las preguntas realizadas se dividen en siete módulos: calidad subjetiva de sueño, duración, perturbaciones del sueño, latencia, uso de medicación para dormir, eficiencia y disfunción diurna ^15^. Estos siete componentes son evaluados y se obtiene un *score* global que puede variar entre 0 y 21 puntos. Si el puntaje es >5, entonces se define como mala calidad de sueño. En el año 2015, este instrumento fue validado para población peruana utilizando una muestra de 4445 personas ^16^.

Dificultad para dormir: fue evaluada a través de la pregunta: *«Durante el último mes, ¿ha tenido dificultad para poder dormir?»*, con tres opciones de respuesta: *«*casi nunca», *«*a veces*»,* y *«*frecuentemente*»*. Para análisis, esta variable se colapsó juntado las dos primeras opciones de respuesta (casi nunca/a veces vs. frecuentemente).

*Variable independiente*

La variable independiente fue estado glicémico, con énfasis en DM2. Esta se definió en base a los estándares de la Asociación Americana de Diabetes (ADA) usando la prueba de tolerancia oral a la glucosa ^17^: aquellos con DM2 fueron los que tenían valores de glucosa plasmática en ayuno ≥126 mg/dL o valores de glucosa postprandial (a las 2 h) ≥200 mg/dL o los que reportaron tener diagnóstico previo realizado por un médico de dicha condición (sí vs. no). Para fines de análisis y basado en reportes previos ^18^, a las personas con presencia de DM2 se les subdividió en dos categorías en base a si tenían o no diagnóstico previo. De esta forma, la variable final presentó tres categorías: sin DM2; con DM2, pero sin diagnóstico previo, y con DM2 y con diagnóstico previo.

*Otras variables*

Las otras variables que se utilizaron en el análisis fueron: sexo (varón vs. mujer); edad (<50 vs. ≥50 años); nivel de educación (<7 años, 7-11 años y ≥12 años); nivel socioeconómico, evaluado a través de las posesiones en la vivienda y luego categorizado en terciles (bajo, medio y alto); si actualmente está trabajando (sí vs. no); tabaquismo diario (sí vs. no); consumo de alcohol (≤1 vez por mes vs. >1 vez por mes); nivel de actividad física, evaluado a través de la versión corta del Cuestionario Internacional de Actividad Física (IPAQ) y categorizado en moderado/alto vs. bajo; índice de masa corporal (normal, sobrepeso y obesidad), definido de acuerdo con puntos de corte tradicionales; e hipertensión arterial (sí vs. no), definido de acuerdo con la presencia de presión arterial sistólica ≥140 mm Hg, o presión arterial diastólica ≥90 mm Hg, o el reporte de diagnóstico previo realizado por un médico.

### Procedimientos

Los procedimientos del estudio original se han detallado previamente ^12^. Brevemente, después de obtener el consentimiento informado, los participantes fueron citados en ayunas para la realización de la prueba de tolerancia oral a la glucosa. Las muestras de sangre fueron obtenidas por personal de salud capacitado. Se extrajo sangre venosa de los participantes, los cuales debían haber permanecido en ayuno entre 8 y 12 horas antes de la toma de la primera muestra, mientras que la segunda muestra fue tomada a las dos horas posingesta de 75 g de glucosa anhidra disuelta en 300 mL de agua ^17^. Los cuestionarios y las mediciones clínicas y antropométricas se realizaron aprovechando el tiempo entre las dos tomas de muestras de sangre. 

Los cuestionarios fueron aplicados mediante encuestas en tabletas, utilizando el *software* Open Data Kit, realizadas por personal de salud capacitado. Los cuestionarios incluyeron información de *scores* de riesgo para DM2, además de otros detalles sociodemográficos y de estilos de vida.

Posteriormente, se realizaron mediciones de talla (usando un tallímetro calibrado), de peso con una balanza de bioimpedancia (TANITA TBF-300A) y circunferencia de la cintura (en cm), medido por triplicado. Asimismo, se midió la presión arterial usando un monitor automático marca OMRON HEM-780 por triplicado, después de 5 min de reposo (con el promedio de las dos últimas mediciones se determinó la presencia de hipertensión arterial).

### Análisis estadístico

Para el análisis se usó el programa STATA versión 16 para Windows (StataCorp, College Station, TX, USA). Primero, se caracterizó a la población de estudio utilizando medias y desviación estándar (DE) para variables numéricas, y proporciones para las variables categóricas. Asimismo, se estimó la prevalencia de duración de sueño (normal, corto y prolongado), mala calidad de sueño, y de dificultad para dormir, así como la prevalencia de DM2 en la población de estudio. Las comparaciones de las distintas variables sociodemográficas y conductuales de acuerdo a la presencia de DM2 y las características del sueño fueron realizadas usando la prueba de chi cuadrado.

Por último, se crearon modelos crudos y ajustados utilizando modelos de regresión de Poisson con varianza robusta para evaluar si existían las asociaciones de interés. Los potenciales confusores se escogieron de acuerdo con un modelo epidemiológico basado en la literatura existente al respecto. Se reportaron razones de prevalencia (RP) e intervalos de confianza al 95% (IC 95%).

### Aspectos éticos

El protocolo del estudio original y los formatos de consentimiento y cuestionarios fueron aprobados por el Comité de Ética de la Universidad Peruana Cayetano Heredia, en Lima, y por el Comité Institucional de Ética de London School of Hygiene and Tropical Medicine, en Londres. Para la realización del presente trabajo los datos fueron codificados para garantizar el anonimato de los participantes. Se obtuvo la aprobación del comité de ética de la Universidad Científica del Sur (137-2020-PRE15). 

## RESULTADOS

### Características de la población de estudio

Se enrolaron 1612 personas, pero 3 (0,2%) participantes no completaron los exámenes de la prueba de tolerancia oral a la glucosa y 5 (0,3%) no tenían datos completos en las variables relacionadas con sueño. Por lo tanto, solo 1604 individuos fueron analizados. La media de edad de toda la muestra fue de 48,2 años (DE: 10,6); 809 (50,3%) fueron mujeres; el 73,5% presentaba sobrepeso u obesidad, y el 67,8% contaba con trabajo al momento del estudio.

### Diabetes *mellitus* tipo 2 y factores asociados

De los 1604 individuos analizados, 175 (10,9%; IC 95%: 9,4% - 12,5%) tuvieron DM2, y de estos últimos, 71 (4,4%) no sabían que tenían dicha condición, mientras que 105 (6,5%) tenían diagnóstico previo. De las variables evaluadas en análisis bivariado, la edad (p<0,001), el nivel de educación (p<0,001), estar actualmente trabajando (p=0,001), el nivel de actividad física (p=0,005), el índice de masa corporal (p=0,023), y la presencia de hipertensión (p<0,001) estuvieron asociadas a DM2 (Tabla 1).

**Tabla 1:** Características de la población del estudio, según estado glicémico.

Característica	Sin DM2	DM2 sin diagnóstico previo	DM2 con diagnóstico previo	Valor de p
	(n = 1429)	(n = 71)	(n = 104)	
Sexo				
Varón	724 (50,7%)	29 (40,9%)	44 (42,3%)	0,081
Mujer	705 (49,3%)	42 (59,1%)	60 (57,7%)	
Edad				
< 50 años	859 (60,1%)	36 (50,7%)	23 (22,1%)	<0,001
≥ 50 años	570 (39,9%)	35 (49,3%)	81 (77,9%)	
Nivel de educación				
< 7 años	438 (30,7%)	27 (38,0%)	52 (50,0%)	<0,001
7 - 11 años	673 (47,1%)	32 (45,1%)	41 (39,4%)	
≥ 12 años	318 (22,2%)	12 (16,9%)	11 (10,6%)	
Nivel socioeconómico				
Bajo	469 (32,8%)	25 (35,2%)	42 (40,4%)	0,430
Medio	496 (34,7%)	20 (28,2%)	33 (31,7%)	
Alto	464 (32,5%)	26 (36,6%)	29 (27,9%)	
Actualmente trabajando				
No	439 (30,7%)	29 (40,9%)	49 (47,1%)	0,001
Sí	990 (69,3%)	42 (59,1%)	55 (52,9%)	
Tabaquismo diario				
No	1345 (94,1%)	69 (97,2%)	98 (94,2%)	0,556
Sí	84 (5,9%)	2 (2,8%)	6 (5,8%)	
Uso de alcohol				
≤ 1 vez por mes	1287 (90,1%)	63 (88,7%)	101 (97,1%)	0,054
>1 vez por mes	142 (9,9%)	8 (11,3%)	3 (2,9%)	
Nivel de actividad física				
Moderado/alto	909 (63,6%)	44 (62,0%)	49 (47,1%)	0,004
Bajo	520 (36,4%)	27 (38,0%)	55 (52,9%)	
Índice de masa corporal				
Normal	388 (27,2%)	11 (15,5%)	26 (25,0%)	0,023
Sobrepeso	625 (43,7%)	28 (39,4%)	52 (50,0%)	
Obeso	416 (29,1%)	32 (45,1%)	26 (25,0%)	
Hipertensión				
No	1084 (75,9%)	46 (64,8%)	58 (57,8%)	<0,001
Sí	345 (24,1%)	25 (35,2%)	46 (44,2%)	

DM2: diabetes *mellitus* tipo 2

### Características del sueño y factores asociados

Respecto a la duración de sueño, la población del estudio reportó dormir en promedio 7,8 h (DE: 1,2). Un 12,0% (IC 95%: 10,5% - 13,7%) de sujetos presentó sueño corto, mientras que 8,2% (IC 95%: 6,9% - 9,6%) presentó sueño prolongado. La edad (p=0,012), el sexo (p<0,001), el nivel de educación (p=0,030), el nivel socioeconómico (p=0,007), estar actualmente trabajando (p<0,001), el tabaquismo diario (p=0,001), el nivel de actividad física (p= 0,003), y el índice de masa corporal (p=0,042) fueron variables asociadas con la duración de sueño en análisis bivariado (Tabla 2).

**Tabla 2:** Características de la población del estudio, según duración del sueño.

Características	Duración de sueño			Valor de p
	Sueño normal	Sueño corto	Sueño largo	
	(n = 1280)	(n = 193)	(n = 131)	
Sexo				
Varón	627 (49,0%)	120 (62,2%)	50 (38,2%)	<0,001
Mujer	653 (51,0%)	73 (37,8%)	81 (61,8%)	
Edad				
< 50 años	756 (59,1%)	95 (49,2%)	67 (51,2%)	0,012
≥ 50 años	524 (40,9%)	98 (50,8%)	65 (48,8%)	
Nivel de educación				
< 7 años	399 (31,2%)	68 (35,2%)	50 (38,2%)	0,030
7 - 11 años	606 (47,3%)	76 (39,4%)	64 (48,9%)	
≥ 12 años	275 (21,5%)	49 (25,4%)	17 (12,9%)	
Nivel socioeconómico				
Bajo	430 (33,6%)	50 (25,9%)	56 (42,8%)	0,007
Medio	450 (35,2%)	66 (34,2%)	33 (25,2%)	
Alto	400 (31,2%)	77 (39,9%)	42 (32,0%)	
Actualmente trabajando				
No	411 (32,1%)	42 (21,8%)	64 (48,9%)	<0,001
Sí	869 (67,9%)	151 (78,2%)	67 (51,1%)	
Tabaquismo diario				
No	1215 (94,9%)	171 (88,6%)	126 (96,2%)	0,001
Sí	65 (5,1%)	22 (11,4%)	5 (3,8%)	
Uso de alcohol				
≤ 1 vez por mes	1160 (90,6%)	170 (88,1%)	121 (92,4%)	0,395
>1 vez por mes	120 (9,4%)	23 (11,9%)	10 (7,6%)	
Nivel de actividad física				
Moderado/alto	803 (62,7%)	133 (68,9%)	66 (50,4%)	0,003
Bajo	477 (37,3%)	60 (31,1%)	65 (49,6%)	
Índice de masa corporal				
Normal	347 (27,1%)	42 (21,8%)	36 (27,5%)	0,042
Sobrepeso	573 (44,8%)	87 (45,1%)	45 (34,4%)	
Obeso	360 (28,1%)	64 (33,1%)	50 (38,1%)	
Hipertensión				
No	953 (74,5%)	142 (73,6%)	93 (71,0%)	0,681
Sí	327 (25,5%)	51 (26,4%)	38 (29,0%)	
Estado glicémico				
No DM2	1141 (89,1%)	173 (89,6%)	115 (87,8%)	0,377
DM2 sin diagnóstico previo	60 (4,7%)	8 (4,2%)	3 (2,3%)	
DM2 con diagnóstico previo	79 (6,2%)	12 (6,2%)	13 (9,9%)	

DM2: diabetes mellitus tipo 2

De otro lado, 3,7% (IC 95%: 2,8% - 4,7%) reportó dificultad para dormir, mientras que 19,5% (IC 95%: 17,5% - 21,5%) de los sujetos tuvieron mala calidad de sueño. En el análisis bivariado, el sexo (p=0,027), la edad (p= 0,019), el nivel de educación (p=0,035), y estar actualmente trabajando (p=0,002) estuvieron asociadas con dificultad para dormir; mientras que la edad (p<0,001), el sexo (p=0,002), el nivel de educación (p<0,001), estar actualmente trabajando (p<0,001) y la presencia de hipertensión (p=0,006)  estuvieron asociadas con mala calidad de sueño (Tabla 3).

**Tabla 3:** Características de la población del estudio, según dificultad para dormir y

Características	Dificultad para dormir		Valor de p	Calidad de sueño		Valor de p
	No	Sí		Buena	Mala	
	(n = 1545)	(n = 59)		(n = 1292)	(n = 312)	
Sexo						
Varón	776 (50,2%)	21 (35,6%)	0,027	683 (52,9%)	114 (36,5%)	<0,001
Mujer	769 (49,8%)	38 (64,4%)		609 (47,1%)	198 (63,5%)	
Edad						
< 50 años	893 (57,8%)	25 (42,4%)	0,019	768 (59,4%)	150 (48,1%)	<0,001
≥ 50 años	652 (42,2%)	34 (57,6%)		524 (40,6%)	162 (51,9%)	
Nivel de educación						
< 7 años	489 (31,7%)	28 (47,5%)	0,035	387 (30,0%)	130 (41,7%)	<0,001
7 - 11 años	726 (47,0%)	20 (33,9%)		622 (48,1%)	124 (39,7%)	
≥ 12 años	330 (21,3%)	11 (18,6%)		283 (21,9%)	58 (18,6%)	
Nivel socioeconómico						
Bajo	514 (33,3%)	22 (37,3%)	0,502	425 (32,9%)	111 (35,6%)	0,665
Medio	533 (34,5%)	16 (27,1%)		446 (34,5%)	103 (33,0%)	
Alto	498 (32,2%)	21 (35,6%)		421 (32,6%)	98 (31,4%)	
Actualmente trabajando						
No	487 (31,5%)	30 (50,9%)	0,002	391 (30,3%)	126 (40,4%)	<0,001
Sí	1058 (68,5%)	29 (49,1%)		901 (69,7%)	186 (59,6%)	
Tabaquismo diario						
No	1456 (94,2%)	56 (94,9%)	0,827	1221 (94,5%)	291 (93,3%)	0,400
Sí	89 (5,8%)	3 (5,1%)		71 (5,5%)	21 (6,7%)	
Uso de alcohol						
≤ 1 vez por mes	1395 (90,3%)	56 (94,9%)	0,235	1168 (90,4%)	283 (90,7%)	0,870
>1 vez por mes	150 (9,7%)	3 (5,1%)		124 (9,6%)	29 (9,3%)	
Nivel de actividad física						
Moderado/alto	966 (62,5%)	36 (61,0%)	0,814	804 (62,2%)	198 (63,5%)	0,687
Bajo	579 (37,5%)	23 (39,0%)		488 (37,8%)	114 (36,5%)	
Índice de masa corporal						
Normal	417 (27,0%)	8 (13,6%)	0,071	358 (27,7%)	67 (21,5%)	0,076
Sobrepeso	675 (43,7%)	30 (50,8%)		561 (43,4%)	144 (46,1%)	
Obeso	453 (29,3%)	21 (35,6%)		373 (28,9%)	101 (32,4%)	
Hipertensión						
No	1149 (74,4%)	39 (66,1%)	0,155	976 (75,5%)	212 (68,0%)	0,006
Sí	396 (25,6%)	20 (33,9%)		316 (24,5%)	100 (32,0%)	
Estado glicémico						
No DM2	1362 (89,4%)	47 (79,6%)	0,004	1165 (90,2%)	264 (84,6%)	0,007
DM2 sin diagnóstico previo	69 (4,5%)	2 (3,4%)		55 (4,2%)	16 (5,1%)	
DM2 con diagnóstico previo	94 (6,1%)	10 (17,0%)		72 (5,6%)	32 (10,3%)	

DM2: diabetes *mellitus *tipo 2

### Asociación entre diabetes mellitus tipo 2 y características del sueño

En el modelo multivariable (Tabla 4), ajustado por edad, sexo, nivel de educación, nivel socioeconómico, estar actualmente trabajando, tabaquismo diario, uso de alcohol, nivel de actividad física y presencia de hipertensión, la presencia de DM2 con diagnóstico previo incrementó la prevalencia de dificultad para dormir (RP = 2,20; IC 95%: 1,13 - 4,27) y de mala calidad de sueño (RP = 1,40; IC 95%: 1,05 - 1,92) comparado contra aquellos sin DM2. De otro lado, no se encontró asociación entre DM2 y duración de sueño.

**Tabla 4:** Asociación entre estado glicémico y sueño: modelos crudos y ajustados.

Características del sueño	Modelo crudo	Modelo ajustado ^a^
	RP (IC 95%)	RP (IC 95%)
Duración de sueño		
Sueño corto vs. normal		
No DM2	1 (Referencia)	1 (Referencia)
DM2 sin diagnóstico previo	0,89 (0,46 - 1,74)	0,91 (0,46 - 1,80)
DM2 con diagnóstico previo	1,00 (0,58 - 1,73)	0,96 (0,55 - 1,65)
Sueño largo vs. normal		
No DM2	1 (Referencia)	1 (Referencia)
DM2 sin diagnóstico previo	0,52 (0,17 - 1,59)	0,44 (0,14 - 1,34)
DM2 con diagnóstico previo	1,54 (0,91 - 2,63)	1,14 (0,68 - 1,94)
Dificultad para dormir		
No DM2	1 (Referencia)	1 (Referencia)
DM2 sin diagnóstico previo	0,86 (0,21 - 3,46)	0,70 (0,17 - 2,85)
DM2 con diagnóstico previo	2,92 (1,52 - 5,62)	2,20 (1,13 - 4,27)
Calidad de sueño		
No DM2	1 (Referencia)	1 (Referencia)
DM2 sin diagnóstico previo	1,22 (0,78 - 1,90)	1,08 (0,70 - 1,67)
DM2 con diagnóstico previo	1,67 (1,22 - 2,27)	1,40 (1,05 - 1,92)

DM2: diabetes *mellitus* tipo 2, RP: razón de prevalencia, IC 95%: intervalo de confianza al 95%.

a Ajustado por edad, sexo, nivel de educación, nivel socioeconómico, actualmente trabajando, tabaquismo diario, uso de alcohol, nivel de actividad física, e hipertensión.

## DISCUSIÓN

De acuerdo con el presente estudio, los individuos con DM2 con diagnóstico previo presentan mayor probabilidad de dificultad para dormir (un incremento del 120%), y mayor prevalencia de mala calidad de sueño (incremento del 40%), comparado con aquellos que no presentaban DM2. La presencia de DM2 sin diagnóstico previo (es decir, aquellos con diagnóstico reciente) no estuvo asociada con ninguna característica del sueño. De otro lado, se encontró que uno de cada diez participantes tenía DM2.

Algunos estudios han encontrado asociación entre DM2 y ciertas características del sueño. Aunque nosotros no encontramos asociación entre DM2 y duración de sueño, una revisión sistemática que solo incluía estudios en China encontró que la prevalencia de sueño corto, definido como aquel <6 horas, fue de 23% en personas con DM2 comparado con 12% en personas saludables ^19^. De otro lado, un estudio usando los datos de nueve países (Finlandia, Polonia, España, China, Ghana, India, México, Rusia, y Sudáfrica) encontró asociación entre DM2 y problemas del sueño en forma global, pero en el análisis por país, la asociación estuvo solo presente en la India ^20^. Este último estudio definió DM2 en base al autorreporte y, por lo tanto, puede asemejarse a nuestro grupo con DM2 y diagnóstico previo. Sin embargo, la variable problemas del sueño no fue evaluada en forma exhaustiva pues solo se hizo usando una pregunta. Otro trabajo encontró que aquellos con DM2 tenían mayores tasas de insomnio, excesiva somnolencia, y solían usar mayor cantidad de hipnóticos que los controles ^21^. Finalmente, un estudio en varones de Suecia reportó mayores dificultades para iniciar y mantener el sueño en pacientes con DM2 que sujetos sin esta condición ^22^. Todos estos resultados conllevan a pensar que DM2 puede alterar ciertas características del sueño, especialmente asociados con la duración y la presencia de dificultades para conciliar el sueño.

Es importante destacar que la DM2, además de causar alteraciones directas del sueño como consecuencia de la nicturia, poliuria, neuropatía diabética y dolor por la neuropatía, también se ha asociado con enfermedades como apnea obstructiva del sueño, hipertensión, complicaciones cardiovasculares, accidentes cerebrovasculares, obesidad, irritabilidad, sed excesiva e incluso depresión, las cuales pueden alterar el sueño ^23^. A su vez, las alteraciones del sueño en las personas diabéticas incrementan la liberación de glucocorticoides produciendo un incremento en la producción de glucosa y un consiguiente ineficiente control glicémico ^24^ ^,^ ^25^.

Aunque en el Perú existe la “Guía de Práctica Clínica para el Diagnóstico, Tratamiento y Control de la Diabetes *Mellitus* tipo 2 en el Primer Nivel de Atención» ^26^, es importante mencionar que actualmente dicha guía no contempla que la población con DM2 podría tener alteraciones del sueño. Nuestro estudio encontró una asociación entre DM2 con diagnóstico previo y mala calidad de sueño, implicando una alta prevalencia de pobre calidad de sueño en aquellos con DM2 a nivel poblacional, para los cuales no se ha detallado un manejo apropiado. Un metaanálisis de estudios de cohorte reportó que las alteraciones de sueño en los pacientes con DM2 conllevan a una serie de complicaciones como pobre control glicémico, intolerancia a la glucosa, y resistencia a insulina ^25^ ^),^ alteración de la homeostasis del sistema inmunológico y enfermedades cardiovasculares ^27^, e incluso aumento de la mortalidad ^28^.

Dado nuestros resultados, sería necesario incluir en las guías actuales, herramientas prácticas que tomen en cuenta preguntas simples para evaluar las características del sueño, puesto que podrían ser de gran utilidad para hacer un tamizaje de las alteraciones de sueño y mejorar el manejo de los casos con DM2. Después de una evaluación inicial, se podrían considerar herramientas más complejas, si fuese necesario. Las interconsultas a las especialidades podrían ser necesarias para garantizar un manejo integral del paciente con DM2.

Finalmente, se identificó que Tumbes es una ciudad con alta prevalencia de DM2 en comparación con el promedio nacional ^9^, y por esto sería necesario identificar y comprender los factores asociados con estas diferencias para diseñar medidas de salud pública orientadas a controlar, prevenir y promocionar la salud de esta población. 

Este es un estudio de base poblacional que utilizó la prueba de tolerancia oral a la glucosa para definir DM2, el cual es considerada la prueba dorada para el diagnóstico de esta condición. A pesar de sus fortalezas, esta investigación presenta algunas limitaciones que merecen resaltarse. Primero, al ser un estudio transversal solo se puede estudiar asociación, pero no causalidad. Segundo, podría existir sesgo de memoria, dado que varios de los factores evaluados son subjetivos y preguntados con respecto al año previo o a los 30 días previos a la fecha de evaluación, a pesar de haber usado escalas validadas como la de Pittsburgh, que presentan una gran fiabilidad ^29^. Asimismo, los resultados no se pueden extrapolar a toda la población peruana ya que para el presente estudio se escogió una región con alta prevalencia de DM2. Tercero, existe la posibilidad de causalidad reversa dado que las alteraciones de las características del sueño también podrían causar DM2 como ha sido descrito en otros trabajos ^30^ ^,^ ^31^. No obstante, las características del sueño fueron evaluadas con respecto a los 30 días previos de la fecha de la entrevista, lo que establece que es probable que la DM2 ocurrió primero. Cuarto, solo se usó una prueba de tolerancia a la glucosa para definir DM2 cuando lo usual, de acuerdo con la ADA, es usar dos pruebas en distintos momentos ^17^. Finalmente, nuestros modelos multivariables fueron ajustados por las variables existentes en la base de datos, dejando de lado otras de importancia como tiempo de enfermedad, presencia de complicaciones (enfermedad coronaria, enfermedad vascular periférica, retinopatía, neuropatías, nefropatías, etc.), hipercolesterolemia, consumo de café, entre otros ^32^ ^-^ ^34^. Sin embargo, nuestros hallazgos están en línea con otros resultados previos.

En conclusión, los individuos con DM2 que tenían diagnóstico previo tuvieron mayor probabilidad de presentar dificultad para dormir y mala calidad de sueño. Nuestros resultados sugieren la necesidad de evaluar en forma periódica las características del sueño en pacientes con DM2.
